# A tolerability assessment of new respiratory protective devices developed for health care personnel: A randomized simulated clinical study

**DOI:** 10.1371/journal.pone.0209559

**Published:** 2019-01-09

**Authors:** Lewis J. Radonovich, Kerri Wizner, Sherri L. LaVela, Martin L. Lee, Kimberly Findley, Patrick Yorio

**Affiliations:** 1 Centers for Disease Control and Prevention, National Institute for Occupational Safety and Health, National Personal Protective Technology Laboratory, Pittsburgh, PA, United States of America; 2 Department of Veterans Affairs, VA Health Services Research and Development, Edward J. Hines, Jr. VA Hospital, Chicago, IL, United States of America; 3 Feinberg School of Medicine, Department of Physical Medicine and Rehabilitation, Northwestern University, Chicago, IL, United States of America; 4 Department of Veterans Affairs Greater Los Angeles Health care System, Los Angeles, CA, United States of America; 5 Department of Biostatistics, University of California, Los Angeles, Los Angeles, CA, United States of America; 6 Department of Veterans Affairs, Center of Innovation on Disability & Rehabilitation Research, North Florida/South Georgia Veterans Health System, Gainesville, FL, United States of America; Uniformed Services University of the Health Sciences, UNITED STATES

## Abstract

**Background:**

U.S. health care personnel (HCP) have reported that some respiratory protective devices (RPD) commonly used in health care have suboptimal tolerability. Between 2012 and 2016, the U.S. National Institute for Occupational Safety and Health, and the Veterans Health Administration collaborated with two respirator manufacturers, Company A and B, to bring new RPD with improved tolerability to the U.S. health care marketplace. The purpose of this study was to compare the tolerability of four new prototype RPD to two models commonly used in U.S. health care delivery.

**Methods:**

A randomized, simulated workplace study was conducted to compare self-reported tolerability of four new prototype RPD (A1, A2, B1, and B2) worn by HCP and two N95 control respirators commonly used in U.S. health care delivery, the 1870 and 1860, manufactured by 3M Corporation. A new survey tool, the Respirator Comfort, Wearing Experience, and Function Instrument (R-COMFI), developed previously in part for the current study, was used as the primary outcome metric. With a maximum total score of 47, lower R-COMFI scores reflected better self-reported tolerability. Poisson regression analyses were used to estimate prototype relative risks compared to controls.

**Results:**

Conducted between 2014 and 2015 in two inpatient care rooms at the North Florida/South Georgia Veterans Health System, among 383 participants who enrolled, 335 (87.5%) completed the study. Mean total R-COMFI scores for the 3M 1870, 3M 1860, and prototypes A1, A2, B1, and B2 were 8.26, 9.36, 5.79, 7.70, 6.09, and 5.71, respectively. Compared to the 3M 1870, total R-COMFI unadjusted relative risks (RR) and 95 percent confidence intervals (CI) were A1 (RR 0.70, CI 0.60, 0.82), A2 (RR 0.93, CI 0.82, 1.06), B1 (RR 0.74, CI 0.64, 0.85), and B2 (RR 0.69, CI 0.60, 0.80). Compared to the 3M 1860, prototype total R-COMFI unadjusted RR and 95 percent CI were A1 (RR 0.62, CI 0.53, 0.72), A2 (RR 0.82, CI 0.73, 0.93), B1 (RR 0.65, CI 0.57, 0.74), and B2 (RR 0.61, CI 0.53, 0.70). Similarly, models adjusted for demographic characteristics showed that prototypes A1, B1, and B2 significantly improved tolerability scores compared to both controls, while prototype A2 was significantly improved compared to the 3M 1860.

**Conclusions:**

Compared to the 3M 1870 and 3M 1860, two RPDs commonly used in U.S. health care delivery, tolerability improved for three of four newly developed prototypes in this simulated workplace study. The R-COMFI tool, used in this study to assess tolerability, should be useful for future comparative studies of RPD.

## Introduction

Health care personnel (HCP) have reported that many respiratory protective devices (RPD) used in U.S. health care delivery have suboptimal tolerability, possibly having a negative influence on the willingness of HCP to wear RPD during patient care [1–6]. To be fully effective, properly fitting RPD must be worn correctly for the duration of exposures, in accordance with Occupational Safety and Health Administration (OSHA) Respiratory Protection Standards [7]. Among the unfavorable characteristics of RPD reported by HCP have been discomfort [1,2,6,8,9], interference with occupational duties [2,6], interference with communication [2,10], heat accumulation behind the mask [1,6], facial irritation [1,6], and breathing resistance [2,6,9]. HCP have requested availability of new RPD on the U.S. market that are tailored to their specific workplace needs. Among the RPD characteristics sought by HCP for health care delivery include improved comfort, improved straps, less interference with breathing, and proper facial fit despite the presence of facial hair [2,6].

To shepherd to the U.S. marketplace new RPD that meet the needs of HCP, the National Institute for Occupational Safety and Health (NIOSH) and the Veterans Health Administration (VHA) co-led a Federal government interagency working group called the Better Respiratory Equipment using Advanced Technologies for Healthcare Employees (Project BREATHE) from 2008 to 2016. This working group previously published a list of 28 idealized and prioritized criteria to be considered for the next generation of RPD for health care delivery, including 10 criteria pertaining to comfort and tolerability: breathing resistance, facial irritation, allergenicity, facial pressure, facial heat, air exchange, moisture management, mass, odor, and prolonged tolerability [3].

In 2012, NIOSH and VHA partnered with two U.S. respirator manufacturers, Companies A and B, to facilitate the development of new RPD for HCP based on the Project BREATHE criteria, seeking improved cost-conscious models that would be sought by health care delivery organizations. In 2014, NIOSH evaluated the physiologic and subjective performance of several candidate devices in a laboratory setting [11]. After further development efforts, four prototype respirators were selected among numerous candidates for further evaluation by VHA. Between 2014 and 2015, the VHA evaluated the subjective tolerability of these four devices in a clinical workplace setting using as the primary outcome metric a new survey tool, the Respirator Comfort, Wearing, Experience, and Function Instrument (R-COMFI), developed and internally validated, in part, for Project BREATHE [12]. The purpose of the current study was to compare the tolerability of the four selected prototypes to two RPD models commonly worn in U.S. health care delivery, using the R-COMFI scores as the primary outcome metric.

## Methods and materials

### Study design

#### Overview

Between September 8, 2014 and May 15, 2015, we conducted a randomized, simulated workplace study in which HCP performed clinically relevant activities in a fully functional inpatient hospital care room. Compared were comfort, general wearing experience, and function of four newly developed prototype RPD to two N95 respirators commonly used in U.S. health care delivery, the 1870 and 1860 N95 respirators, manufactured by the 3M Corporation (St. Paul, MN). The study was conducted at the North Florida/South Georgia Veterans Health System (Gainesville, FL).

Prior to participant enrollment, the study was approved by the University of Florida Institutional Review Board, Protocol #201300693 and the North Florida/South Georgia Veterans Health System Research and Development Committee, Protocol #201300693. Each participant signed IRB-approved VHA informed consent. Following study completion, a de-identified dataset of the results was provided to the National Personal Protective Technology Laboratory at NIOSH. Prior to conducting analyses, the study was exempted by the NIOSH Human Subjects Research Board, Protocol # 17-NPPTL-01XM.

#### Setting

**Fig 1:**
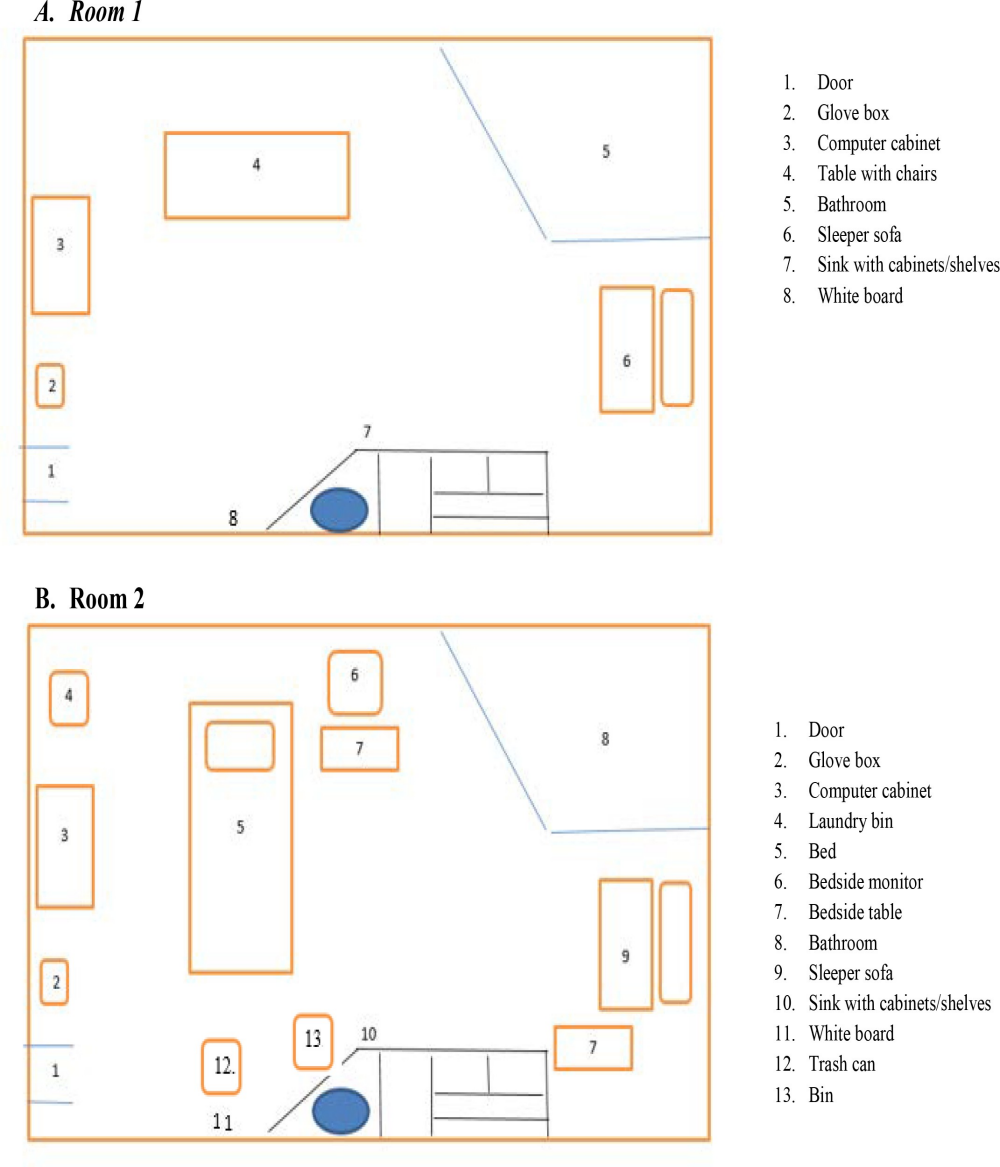
Simulated workplace setting, two single occupancy hospital rooms.

This study was conducted in two single occupancy patient care rooms, both measuring 8’1” x 20’3/8”, in a 240 bed tertiary care hospital. Performed in Room 1 (Fig 1A) were informed consent, randomized RPD assignment, donning of respirators and other protective equipment, a five-minute acclimation period, fit testing, and two concentration activities. All other activities occurred in Room 2, a patient care room, equipped with a hospital bed, bedside table and monitor, computer, sofa, sink, and other patient- and study-related equipment (Fig 1B).

#### Participants

Three hundred eighty-three participants, HCP employed by the North Florida/South Georgia Veterans Health System (Gainesville, FL) and/or Shands Hospital at the University of Florida (Gainesville, FL), were recruited (Table 1).

**Table 1:** Demographic characteristics of study participants and arm assignments.

			Controls		Prototypes			
		Total^b^	1	2	A1	A2	B1	B2
**Number of Participants (%)**^a^		335	53 (15.8)	58 (17.3)	38 (11.3)	67 (20.0)	64 (19.1)	55 (16.4)
**Gender (%)**	Male	87 (26.0)	16 (30.2)	10 (17.2)	6 (15.8)	17 (25.4)	20 (31.3)	18 (32.7)
	Female	248 (74.0)	37 (69.8)	48 (82.8)	32 (84.2)	50 (74.6)	44 (68.8)	37 (67.3)
**Age (%)**	≤25	41 (12.3)	12 (22.6)	4 (7.0)	5 (13.2)	6 (9.0)	9 (14.1)	5 (9.1)
	26–49	206 (61.7)	28 (52.8)	37 (64.9)	23 (60.5)	46 (68.7)	41 (64.1)	31 (56.4)
	>50	87 (26.0)	13 (24.5)	16 (28.0)	10 (26.3)	15 (22.4)	14 (21.9)	19 (34.5)
**Job title (%)**	Nurses or health care assistants	233 (69.8)	37 (69.8)	40 (69.0)	30 (79.0)	45 (67.2)	44 (70.0)	37 (67.3)
	Primary care provider (e.g., physician, nurse practitioner)	47 (14.0)	4 (7.6)	7 (12.1)	4 (10.5)	13 (19.4)	8 (12.7)	11 (20.0)
	Respiratory therapist	18 (5.4)	4 (7.6)	6 (10.3)	2 (5.3)	2 (3.0)	2 (3.2)	2 (3.6)
	Other	36 (10.7)	8 (15.1)	5 (8.6)	2 (5.3)	7 (10.4)	9 (14.1)	5 (9.1)
**Mean weekly hours worked**		41.3	38.2	41.4	39.3	44.7	40.0	44.0
**Mean weekly patient contact hours**		32.1	31.2	31.5	34.7	34.2	31.1	30.1

^a^Participants who passed an OSHA-accepted qualitative fit test are shown (n = 335);

^b^Where summative percentages do not equal 100, participants did not respond to all questions

Inclusion criteria were (a) previously receiving medical clearance to wear a filtering facepiece respirator (FFR) commensurate with regulations issued by OSHA, and (b) passing an OSHA-accepted qualitative fit test while wearing the RPD to which the participant was randomized [7]. Participants were excluded from participation if they exhibited (a) a health condition that precluded wearing an RPD, (b) physical characteristics that may have interfered with the ability to obtain an adequate facial seal during fit testing, (c) pregnancy, or (d) any condition or issue placing the participant at undue risk of harm or interfering with data integrity, as determined by the principal investigator.

### Procedures

**Fig 2:**
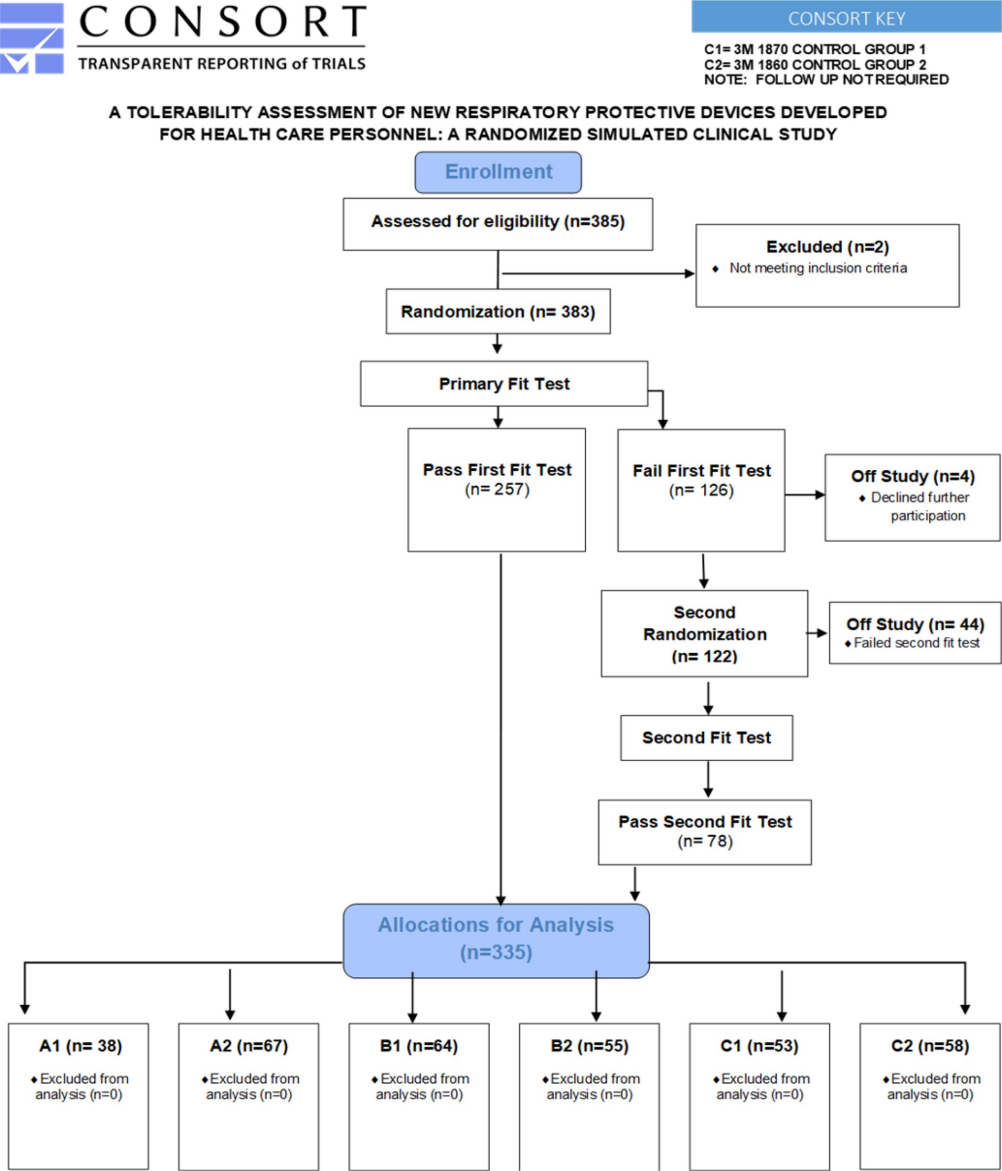
CONSORT flow diagram.

Each eligible participant was randomized, using a random number generator, to wear one of six RPD: prototypes A1, A2; B1, B2, control 1, or control 2 (Table 2). Each participant was permitted to wear only one respirator during one test session; to preclude habituation bias [13], participants did *not* engage in repeated test sessions (Fig 2).

**Table 2:** Respiratory protective devices evaluated^a^.

**Company A Prototype Respirator 1 (A1)**	A filtering facepiece respirator, available in small and medium / large sizes, with a curved, horizontally positioned, oblong shaped, plastic frame that houses a central filter panel, to which is attached a chin panel and a less permeable nasal panel with adjustable aluminum nasal bar, and an adjustable single piece strap.
**Company A Prototype Respirator 2 (A2)**	A reusable filtering facepiece respirator/elastomeric respirator hybrid available in small, medium and large sizes, comprised of a pliable, opaque silicone facemask with a centrally located, vertically positioned, parabolic-shaped, replaceable filter and filter housing with a single piece elasticized harness.
**Company B Prototype Respirator 1 (B1)**	A disposable soft, cup shaped filtering facepiece respirator model available in small and standard sizes with two non-adjustable elasticized straps and a pliable metal nose bar.
**Company B Prototype Respirator 2 (B2)**	A disposable pliable, V-shaped, pleated filtering facepiece respirator model available in small and standard sizes with two non-adjustable elasticized straps and a pliable metal nose bar.
**Control Respirators**	Two commercially-available N95 filtering facepiece respirator models (3M 1860, 3M 1870; 3M Company, St. Paul, MN), are among the most commonly used RPD in U.S. health care ^14^.

^a^Adapted and reprinted with permission from the Journal of the International Society for Respiratory Protection ^11^.

#### Respirators

Four prototype RPDs developed by two private manufacturers (company A and B) in collaboration with NIOSH and VHA were utilized (Table 2). The four concept-level prototypes met filtration test requirements of ≥ 95% efficiency, none were equipped with an exhalation valve, and none represented final design lockdown nor the refinement that would be expected of a mass production sample. Three (A1, B1, B2) were filtering facepieces and one (A2) was an elastomeric half-mask respirator. Two N95 models commonly used in U.S. health care delivery [14], the 3M 1860 and 3M 1870, were used as controls.

#### Fit testing

Following informed consent and intervention assignment, OSHA-accepted qualitative fit testing was conducted using Bitrex, except for one participant who was tested with saccharin due to a previous adverse reaction to Bitrex. A user seal check was performed, followed by a five minute acclimation period while wearing the respirator. Participants who failed fit testing on the first RPD to which they were initially randomized were randomized again and fit tested for a second RPD. Participants who failed both an initial and secondary fit tests were excluded from further participation (Fig 2).

#### Simulated workplace test sessions

Participants performed simulated HCP workplace tasks designed to last approximately one hour. The research assistant played the role of a simulated patient when patient assessment activities were performed.

#### Test session initiation

After donning nitrile gloves (model XTRA, Kimberly-Clark, Irving, Texas), an isolation gown (model NON-27-SMS-2, Medline Industries, Northfield, IL) and safety glasses (model 11000–500, Fisher Scientific, Hampton, NH), if they were not already wearing corrective eyeglasses, participants were instructed to don their assigned RPD using manufacturers’ written instructions as a guide. Following a user seal check ^7^, the research assistant started timing the length of the test session.

#### Test session activities and tasks

Participants completed a series of clinically relevant simulated workplace activities (Table 3).

**Table 3:** Simulated workplace activities, tasks, and approximate timeframe.

	Performance Requirements	Clinical Relevance	Room Performed	Room Location and Body Posture	Approximate Duration (minutes)
**Donning protective equipment**					
Put on gown	Upper extremeity and trunk movement	Donning protective equipment	1	Standing at table	0.5–1.0
Put on safety glasses	Upper extremity and head movement	Donning protective equipment	1	Standing at table	0.3–0.5
Put on respirator	Upper extremity and head movement	Donning protective equipment	1	Standing at table	1.0–5.0
**Concentration**					
Assemble Jig-saw puzzle assembly (100 pieces)^a^	Concentration, fine motor skills	Problem solving	1	Seated at table	8.0–12
Sort, match, and assemble colored caps and vials 50 pieces each); place vials in color-matched container	Concentration, fine motor skills	Organization	1	Seated at table	3.0–5.0
**Hand hygiene and donning gloves**					
Wash hands with soap^b^ and water; dry hands with paper towels^c^	Standing & bending at trunk	Performing hand hygiene	2	Standing at sink	1.0–2.0
Don nitrile exam gloves	Standing, reaching, upper extremity use	Donning protective equipment	2	Standing next to table	0.5–1.0
**Patient Interaction**					
Introduce self to patient and explain assessment activities to be conducted	Verbal & nonverbal communication	Establishing patient rapport	2	Standing at bedside	0.5–1.0
Auscultate right antecubital systolic and diastolic blood pressure using manual blood pressure cuff^d^, sphygmomaneter^d^, and stethescope^e^	Bending at trunk, upper extremity use	Auscultation	2	Standing at bedside	1.0–2.0
Palpate radial pulse and determine pulse rate using wall-mounted clock	Bending at trunk, upper extremity use	Palpation	2	Standing at bedside	0.3–0.5
Determine respiratory rate	Counting, calculation	Observation	2	Standing at bedside	0.3–0.5
Measure tympanic membrane temperature with a digital thermometer^f^	Upper extremity use	Utilizing digital device	2	Standing at bedside	0.3–0.5
Transcribe vital signs on notepad	Hand writing	Recording vital signs	2	Standing at bedside	0.5–1.0
Enter vital sign data into patient assessment template using desktop computer^g^^,^^h^	Typing	Performing computer entry	2	Seated at desk	1–1.5
Transcribe information (fabricated by research assistant) from notepad to wall-mounted white board: today’s date, room number, telephone number, daily goals, anticipated discharge date, names of attending physician, nurse technician, and case manager	Hand writing, reaching	Transcribing data	2	Standing at white board	1.0–3.0
**Ergonomic and Exertional Activities**					
Switch on bedside monitor^i^, wait for it to illuminate, switch off bedside monitor	Reaching	Performing low intensity exertion	2	Standing at bedside	0.5–1.0
Lift 5 lb. weight from surface of bed and place it on the bedside table	Reaching, Lifting	Performing Low intensity exertion	2	Standing at bedside	0.3–0.5
Squat next to the bed and read out-loud a phrase^j^ printed on a placard that is located on the bed frame prior to standing again.	Squatting, reading, talking	Performing low intensity exertion	2	Standing at bedside	03–0.5
Lift 2 lb. weight from surface of bedside table, walk 4 feet to the bookshelf, and place weight on the bookshelf	Lifting, ambulating, reaching	Performing low intensity exertion	2	Standing at bedside	0.5–1.0
Walk 7 feet to bookshelf, obtain water pitcher from bedside stand, walk 9 feet to sink, partially fill water pitcher (1200 cc), walk 9 feet to beside stand, pour water into a cup (240cc) on bedside stand, walk 9 feet to patient seated on couch, hand cup to patient	Lifting, pouring, reaching	Performing low intensity exertion	2	Standing at bedside	1.0–1.5
Walk 7 feet to patient, remove bed sheets, walk 4 feet to laundry bin, place sheets in the laundry bin	Reaching, lifting, ambulating	Performing moderate intensity exertion	2	Standing at bedside	2.0–4.0
Walk 17 feet to wall-mounted cabinet, open cabinet, obtain clean sheets from within cabinet, walk 13 to bedside, remake the bed	Reaching, lifting, ambulating	Performing moderate intensity exertion	2	Ambulating between bed and cabinet	2.0–4.0
**Doffing Protective Equipment and Hand Hygiene**					
Walk to biotrash can, remove exam gloves, discard gloves	Upper extremity use	Doffing protective equipment	2	Ambulating between bed and trash can	0.5–1.0
Remove safety goggles (if applicable) and place in bin	Upper extremity use	Doffing protective equipment	2	Standing at bin	0.25–0.5
Remove isolation gown and discard in biotrash	Upper extremity use	Doffing protective equipment	2	Standing at trash	0.5–1.0
Remove respirator, discard in biotrash (if disposable), place in bin (if reusable)	Upper extremity use	Doffing protective equipment	2	Standing at bin	0.5–1.0
Wash hands with soap and water; dry hands with paper towels	Bending at trunk, upper extremity use	Performing hand hygiene	2	Standing at sink	1.0–2.0

^a^Pink Panther, Golden Puzzles (Racine, WI) measuring 12 x 15 inches or Elmer Fudd, Whitman Puzzles (Poughkeepsie, NY) measuring 14 x 18 inches

^b^Steris (Mentor, OH)

^c^Kimberly Clark (Dallas, TX)

^d^Welch Allyn (Skaneateles, NY), model DS45-11

^e^Central Association for the Blind (Utica, NY), model 6515-00-NIB-0115

^f^Medline Industries (Canton, OH), model MDS9607

^g^Dell (Round Rock, TX)

^h^Simulated patient data was deleted after each test session

^i^Phillips Corporation (Hanover, MD)

^j^**“**Jack be nimble. Jack be quick. Jack jump over the candlestick. Jack be nimble. Jack be spry. Jack jump over the apple pie. Jack be nimble. Jack jump high. Jack fly up into the sky.”

Abbreviations:

cc (cubic centimeters)

#### Test session completion and survey

**Fig 3:**
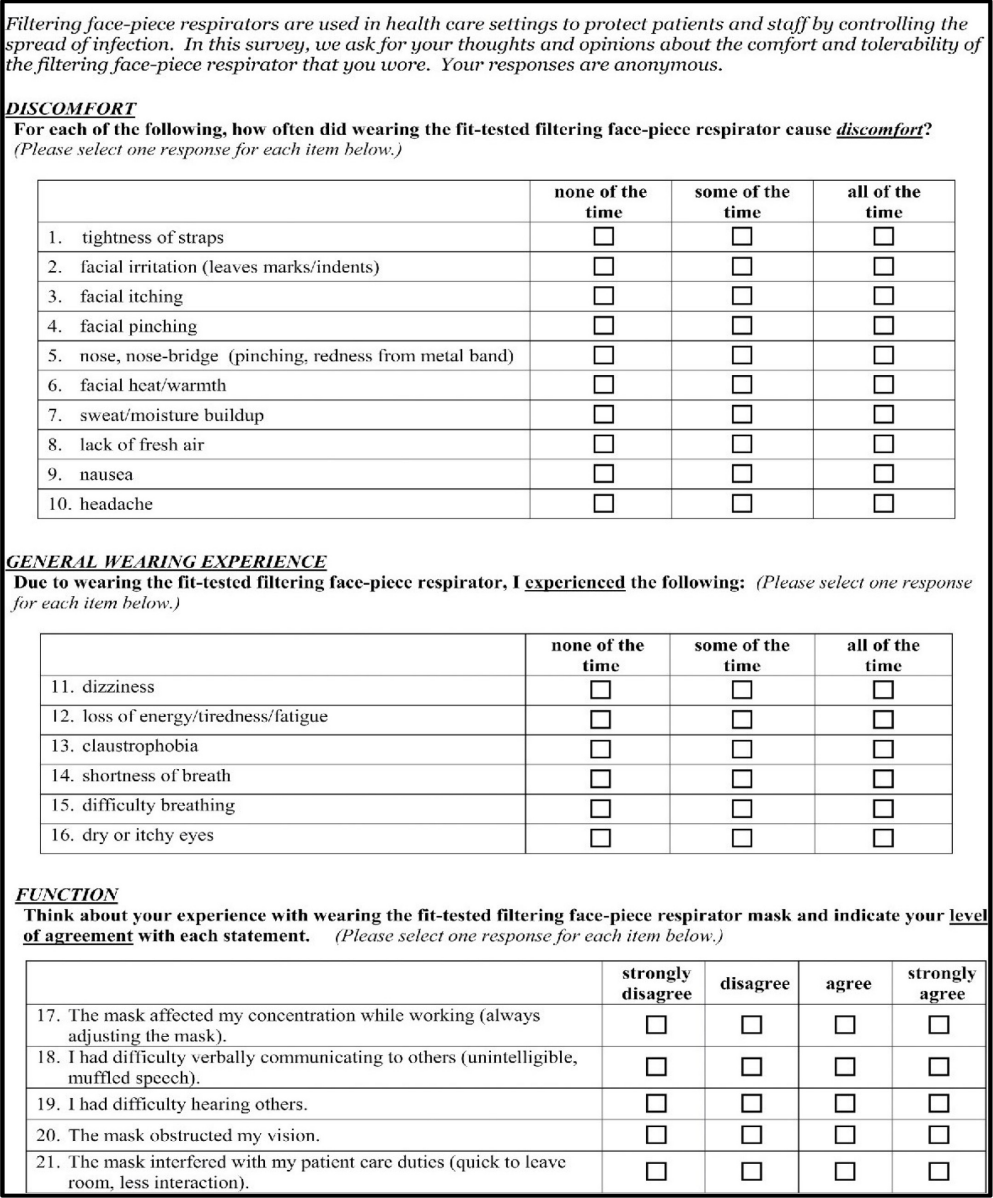
The respirator comfort, wearing experience, and function instrument (R-COMFI) survey^a^. ^a^Reprinted with permission from the Journal of Occupational and Environmental Hygiene (JOEH) [LaVela].

After the research assistant recorded the time of test session completion, participants were instructed to be seated at the Table in Room 2 to complete the data collection instrument, the R-COMFI (Fig 3), that was previously developed, internally validated, and described [12]. In brief, participants answered a series of questions about discomfort, general wearing experience, and function using likert-type response options in which each response was weighted equally. Scores were obtained for the three subscales and summed for a total score. For the discomfort and general wearing experience subscales, possible responses included “none of the time” (zero points), “some of the time” (one point) or “all of the time” (two points). For the function subscale, possible responses included strongly disagree (zero points), disagree (one point), agree (two points), or strongly agree (three points). The range of possible scores was zero to 20, zero to 12, and zero to 15 for the discomfort, general wearing experience, and function subscales, respectively. The total maximum R-COMFI score was 47 in which *lower* scores reflected *better* tolerability.

### Statistical design and analyses

Assuming a global significance level of 5% and power at least 80%, sample size was estimated to be 56 individuals per group (336 in total) to detect a minimum difference of one standard deviation on the total R-COMFI score between control and prototype. A six group block randomized design was used in which each participant wore only one of six respirators to avoid habituation bias and to develop statistical models that did not violate the assumption of independence between observations. Statistical analyses were performed using SAS Institute Inc., SAS 9.1.3 (Cary, NC). Mean R-COMFI subscales (discomfort, general wearing experience, and function) were summed to calculate mean total scores.

The resulting distribution of the collective individual R-COMFI scores deviated from normality (Shapiro-Wilk <0.05) and took the form of a non-negative, positively skewed, integer distribution. Using a regression analytical framework to estimate the mean difference in R-COMFI scores between the prototypes and controls, the fit of a Poisson distribution was examined in relation to the Normal distribution. Consistent with the visual appearance of the data and the results of the Shapiro-Wilk test, the Poisson distribution was found to provide better fit through Akaike’s Information Criterion, Bayesian Information Criterion, and log likelihood values.

Differences in R-COMFI scores were examined using univariate Poisson regression to compare the tolerability of each prototype to both controls. Multivariate Poisson regression was also used to examine differences in R-COMFI scores among the respirator types in relation to demographic characteristics, including the categorical variables gender (male, female), categorical age (≤25, 26–49, ≥50), and job type (nurses and other healthcare assistants, primary care providers, respiratory therapists, and other) and the continuous variables, average weekly hours worked and average weekly hours of patient contact. R-COMFI scores were collected for each of the respirators and aligned with each of the prototypes and controls in the dataset using a categorical variable. This categorical variable was entered into the regression models with the controls as the reference group. This allowed for the comparison of R-COMFI scores for each of the prototypes with each of the control respirators. Differences between each of the prototypes and the each of the controls were examined individually for the total R-COMFI score, discomfort, general wearing experience, and function. Wald *χ*^2^ p-values <0.05 were considered statistically significant. Tolerability was estimated by calculating relative risks (RR) in which each prototype was compared to each control using total R-COMFI scores as the primary outcome.

## Results

Three hundred eighty-three participants were recruited. Demographic characteristics of the participants who were eligible for randomization were similar across study arms (Table 1). Forty-four (11.5%) participants were unable to pass either of two fit tests and were excluded from further participation, including 14 (3.7%), four (1.0%), three (0.8%), seven (1.8%) and 16 (4.0%) assigned to prototype A1, A2, B1, B2, and controls respectively (Fig 2). Four participants declined secondary fit test and were excluded from further participation. Accordingly, the passing rates were 73.1% for prototype A1, 94.4% for prototype A2, 94.1% for prototype B1, 88.7% for prototype B2 and 85.4% for the combined control groups.

Three hundred thirty-five (87.5%) completed the study. Participants were mostly female (74%), aged 26 to 49 (62%), nurses and health care assistants (70%), and worked an average of 41.5 hours weekly, with an average of 32.1 hours of weekly patient contact (Table 1).

Total mean R-COMFI scores for the 3M 1870, 3M 1860, and prototypes A1, A2, B1, and B2 were 8.26, 9.36, 5.79, 7.70, 6.09, and 5.71, respectively (Table 4). Compared to the 1870, prototype total R-COMFI unadjusted relative risks (RR) and 95 percent confidence intervals (CI) were: A1 (RR 0.70, CI 0.60, 0.82), A2 (RR 0.93, CI 0.82, 1.06), B1 (RR 0.74, CI 0.64, 0.85), and B2 (RR 0.69, CI 0.60, 0.80). Compared to the 1860, prototype total R-COMFI unadjusted RR and 95 percent CI were: A1 (RR 0.62, CI 0.53, 0.72), A2 (RR 0.82, CI 0.73, 0.93), B1 (RR 0.0.65, CI 0.57, 0.74), and B2 (RR 0.61, CI 0.53, 0.70). As reflected in Table 4, all comparisons were significant except in the comparison of prototype A2 with the 1870.

**Table 4:** Total R-COMFI scores^a^ for prototype respirators compared to control respirators.

				Unadjusted Relative Risk^b^				Adjusted Relative Risk^b^			
	Respirator Model	Number of Participants (n)	Mean R-COMFI Score	Point Estimate^c^^,^^d^	Wald 95% Confidence Intervals		P-value	Point Estimate^c^^,^^e^	Wald 95% Confidence Intervals		P-value
**Control 1**	3M 1870	53	8.26	1				1			
**Prototypes**	A1	38	5.79	0.7	0.60	0.82	<0.001	0.74	0.63	0.88	<0.001
	A2	67	7.70	0.93	0.82	1.06	0.28	1.01	0.88	1.15	0.939
	B1	64	6.09	0.74	0.64	0.85	< .001	0.75	0.65	0.86	<0.001
	B2	55	5.71	0.69	0.60	0.8	<0.001	0.79	0.68	0.91	0.002
**Control 2**	3M 1860	58	9.36	1				1			
**Prototypes**	A1	38	5.79	0.62	0.53	0.72	<0.001	0.6	0.51	0.70	<0.001
	A2	67	7.70	0.82	0.73	0.93	0.01	0.83	0.73	0.94	0.003
	B1	64	6.09	0.65	0.57	0.74	<0.001	0.61	0.54	0.70	<0.001
	B2	55	5.71	0.61	0.53	0.7	<0.001	0.64	0.55	0.73	<0.001

^a^ Total possible score 47, where lower values represent better tolerability

^b^Relative risk is represented by Exp(B) in Poisson regression

^c^Point estimate (maximum likelihood estimation) assuming a Poisson (non-normal) distribution.

^d^Bivariate Poisson regression

^e^Multivariate Poisson regression

Mean discomfort subscale scores for the 3M 1870, 3M 1860, and prototypes A1, A2, B1, and B2 were 4.62, 4.98, 3.71, 3.97, 3.27, 3.13, respectively (Table 5). Compared to the 1870, prototype discomfort unadjusted RR and 95 percent CI were: A1 (RR 0.80, CI 0.65, 0.99), A2 (RR 0.86, CI 0.72, 1.02), B1 (RR 0.71, CI 0.59, 0.85), and B2 (RR 0.68, CI 0.56, 0.82). Compared to the 1860, prototype discomfort unadjusted RR and 95 percent CI were: A1 (RR 0.75, CI 0.61, 0.91), A2 (RR 0.80, CI 0.68, 0.94), B1 (RR 0.66, CI 0.55, 0.78), and B2 (RR 0.63, CI 0.52, 0.76).

**Table 5:** Subscale R-COMFI scores^a^ for prototype respirators compared to control respirators.

				Unadjusted Relative Risk^b^				Adjusted Relative Risk^b^			
	Respirator Model	Number of Participants (n)	Mean R-COMFI Score	Point Estimate^c^^,^^d^	Wald 95% Confidence Intervals		P-value	Point Estimate^c^^,^^e^	Wald 95% Confidence Intervals		P-value
	**Discomfort**										
**Control 1**	3M 1870	53	4.62	1				1			
**Prototypes**	A1	38	3.71	0.80	0.65	0.99	0.04	0.84	0.68	1.04	0.11
	A2	67	3.97	0.86	0.72	1.02	0.09	0.9	0.76	1.08	0.26
	B1	64	3.27	0.71	0.59	0.85	<0.001	0.71	5.90	0.86	<0.001
	B2	55	3.13	0.68	0.56	0.82	<0.001	0.75	0.61	0.91	0.005
**Control 2**	3M 1860	58	4.98	1				1			
**Prototypes**	A1	38	3.71	0.75	0.61	0.91	0.004	0.70	0.57	0.86	< .001
	A2	67	3.97	0.80	0.68	0.94	0.008	0.77	0.65	0.92	0.003
	B1	64	3.27	0.66	0.55	0.78	<0.001	0.6	0.50	0.72	<0.001
	B2	55	3.13	0.63	0.52	0.76	<0.001	0.63	0.52	0.77	< .001
	**Wearing Experience**										
**Control 1**	3M 1870	53	1.11	1				1			
**Prototypes**	A1	38	0.71	0.64	0.41	1.01	0.05	0.63	0.40	1.01	0.05
	A2	67	1.09	0.98	0.69	1.38	0.90	1.12	0.78	1.60	0.54
	B1	64	0.73	0.66	0.45	0.97	0.03	0.65	0.44	0.96	0.03
	B2	55	0.93	0.83	0.57	1.21	0.34	1.13	0.76	1.66	0.55
**Control 2**	3M 1860	58	1.40	1				1			
**Prototypes**	A1	38	0.71	0.51	0.33	0.79	0.002	0.46	0.29	0.71	0.001
	A2	67	1.09	0.78	0.57	1.07	0.12	0.46	0.29	0.71	0.24
	B1	64	0.73	0.53	0.37	0.75	<0.001	0.47	0.32	0.69	<0.001
	B2	55	0.93	0.66	0.47	0.94	0.02	0.8	0.56	1.14	0.22
	**Function**										
**Control 1**	3M 1870	53	2.52	1				1			
**Prototypes**	A1	38	1.37	0.54	0.39	0.75	<0.001	0.54	0.44	0.84	0.002
	A2	67	2.64	1.05	0.84	1.31	0.70	1.05	0.91	1.46	0.23
	B1	64	2.09	0.83	0.65	1.05	0.12	0.83	0.67	1.09	0.20
	B2	55	1.66	0.65	0.50	0.85	0.002	0.65	0.56	0.97	0.03
**Control 2**	3M 1860	58	2.98	1				1			
**Prototypes**	A1	38	1.37	0.46	0.34	0.63	<0.001	0.46	0.35	0.67	< .001
	A2	67	2.64	0.89	0.72	1.09	0.26	0.89	0.75	1.16	0.54
	B1	64	2.09	0.70	0.56	0.88	0.002	0.7	0.55	0.89	0.003
	B2	55	1.66	0.56	0.43	0.72	<0.001	0.56	0.45	0.76	< .001

^a^ Total possible score 20 for discomfort, 12 for wearing experience, 15 for function, where lower values represent better tolerability

^b^Relative risk is represented by Exp(B) in Poisson regression

^c^Point estimate (maximum likelihood estimation) assuming a Poisson (non-normal) distribution.

^d^Bivariate Poisson regression

^e^Multivariate Poisson regression

Mean wearing experience subscale scores for the 3M 1870, 3M 1860, and prototypes A1, A2, B1, and B2 were 1.11, 1.40, 0.71, 1.09, 0.73, and 0.93, respectively (Table 5). Compared to the 1870, prototype wearing experience unadjusted RR and 95 percent CI were: A1 (RR 0.64, CI 0.41, 0.101), A2 (RR 0.98, CI 0.69, 1.38), B1 (RR 0.66, CI 0.45, 0.97), and B2 (RR 0.83, CI 0.57, 1.21). Compared to the 1860, prototype wearing experience unadjusted RR and 95 percent CI were: A1 (RR 0.51, CI 0.33, 0.79), A2 (RR 0.78, CI 0.57, 1.07), B1 (RR 0.53, CI 0.37, 0.75), and B2 (RR 0.66, CI 0.47, 0.94).

Mean function subscale scores for the 3M 1870, 3M 1860, and prototypes A1, A2, B1, and B2 were 2.52, 2.98, 1.37, 2.64, 2.09, and 1.66, respectively (Table 5). Compared to the 1870, prototype function unadjusted RR and 95 percent CI were: A1 (RR 0.54, CI 0.39, 0.75), A2 (RR 1.05, CI 0.84, 1.31), B1 (RR 0.83, CI 0.65, 1.05), and B2 (RR 0.65, CI 0.50, 0.85). Compared to the 1860, function unadjusted RR and 95 percent CI were: A1 (RR 0.46, CI 0.34, 0.63), A2 (RR 0.89, CI 0.72, 1.09), B1 (RR 0.70, CI 0.56, 0.88), and B2 (RR 0.56, CI 0.43, 0.72).

Using Poisson regression to adjust for demographic characteristics, prototypes A1, B1, and B2 received improved total R-COMFI tolerability scores compared to both controls, while prototype A2 was improved compared to one of the controls, the 3M 1860 (Table 4). Adjusted and unadjusted subscale results were also similar (Table 5); only two scores shifted from marginally significant to non-significant: prototype A1 compared to the 3M 1870 on the discomfort subscale and prototype B2 compared to the 3M 1860 on the wearing experience subscale. Considered together, the adjusted models showed that total R-COMFI scores were significantly predicted by occupation, in which respiratory therapists reported approximately 30 percent lower scores (better tolerability) than nurses; age, in which participants aged 26 to 64 reported 30 to 50 percent lower scores than those aged ≤25; and average weekly hours worked, in which each hour increase was associated with one percent decrease in scores. Participant gender and average weekly patient contact hours were not significant predictors of total R-COMFI scores. No study related serious adverse events were reported. One participant who experienced claustrophobia during fit testing was withdrawn.

## Discussion

U.S. health care personnel have sought, and would benefit from, RPD that are better tolerated in the context of patient care and do not interfere with occupational duties [1,2,4–6,8–10]. After working with two U.S. RPD manufacturers to develop four new prototype respirators designed to meet the needs of HCP, we evaluated the tolerability of each prototype by assessing comfort, general wearing experience, and function using a new instrument, the R-COMFI, in a randomized, simulated workplace study. Compared to the 3M 1870 and 3M 1860, two commonly used RPD in U.S. health care, three of four newly developed prototype RPD received improved total tolerability scores. The fourth prototype received improved total tolerability scores compared to the 3M 1860, but not the 3M 1870. No significant differences in total R-COMFI scores were found between the unadjusted and adjusted models.

On the discomfort subscale, all four prototypes showed improvement compared to the 3M 1860. Compared to the 3M 1870, prototypes B1 and B2 showed improvement; however, model A2 showed no improvement and prototype A1 shifted from a marginal pre-defined level of significance in the unadjusted model to non-significance in the adjusted model. While both companies were able to make substantial improvement in comfort with their FFR prototypes, the hybrid elastomeric prototype made by Company A showed little if any detectable improvement in this study, perhaps a reflection of a unique design that could be refined in the future.

Regarding the wearing experience subscale, prototypes A1 and B1 showed improvement in both unadjusted and adjusted models compared to the 3M 1870 and the 3M 1860, although A1 was marginally significant compared to the 3M 1870. The improvement in B1 may have been related, in part, to familiarity; cup shaped devices like prototype B1 are the most commonly used RPD in U.S. health care [14].

On the function subscale, prototypes A1, B1, and B2 showed improvement in both unadjusted and adjusted models compared to the 3M 1860. Compared to the 3M 1870, prototypes A1 and B2 demonstrated improvement in both models, although prototypes A2 and B1 showed no improvement. Lack of improvement with prototype A2, the hybrid elastomeric respirator, is not surprising given that elastomeric respirators, in general, interfere more with speech intelligibility than FFRs [10]. Interference with communication and occupational duties are among the most common reasons cited for lack of adherence to respiratory protection guidance [1,2,6]. Additional development efforts emphasizing improvement in this characteristic may be beneficial to worker safety [3].

While there is no widely accepted definition of clinical significance, using 25% improvement (relative risk ≤ 0.75) in tolerability as a suitable threshold [15], prototypes A1 and B1 surpassed this level for total R-COMFI scores compared to both controls, and prototype B2 surpassed this level compared to the 3M 1860 control for the function subscale. The higher (less tolerable) scores for wearing experience and function of prototype A2, the hybrid elastomeric, primarily prevented it from surpassing this threshold.

The demographic factors significantly predictive of better tolerability included working as a respiratory therapist, higher age, and higher average weekly hours worked, and may suggest that familiarity with wearing respiratory protection is an important determinant of tolerability. For example, compared to a 2010 HCP tolerability study [1] in which participants worked in a variety of practice settings for eight hour work shifts, participants in a similar 2013 study [16] comprised of participants who worked in an intensive care unit setting for 12-hour work shifts, tended to report better RPD tolerability.

Although prototype A2, the hybrid elastomeric respirator, demonstrated minimal tolerability improvement in our study, it may still hold promise in health care settings, pending refinement. During a hypothetical influenza pandemic, several billion N95 respirators may be needed to protect HCP who serve on the front lines of patient care [17], orders of magnitude less than the number held by acute care hospitals in the U.S. [18]. To conserve N95 and ensure HCP have sufficient numbers of RPD to care for large surges of ill patients, reusable respirators may be necessary [19–22].

The R-COMFI is the first internally validated, comprehensive, and psychometrically sound survey instrument that measures comfort, wearing experience, and function among HCP wearing RPD while performing typical health care tasks. This study represents the first time the R-COMFI, developed for filtering facepiece respirators, was evaluated for external validity in a simulated workplace study. While the *mean* R-COMFI scores reported by participants were generally low, representing six to 25 percent of possible values on the R-COMFI measurement scales, the *range* of values reported by participants utilized 66 to 100 percent of the four scales. The precision of the R-COMFI may be improved by revising the 21 criteria to be more contextually sensitive, especially in settings where RPD are worn for relatively brief periods and routine health care tasks are performed. The R-COMFI validity has not yet been evaluated in settings where prolonged RPD wearing periods are necessary or in situations where more complex medical procedures are performed [23]. As a new tool, the R-COMFI should be helpful in future efforts to evaluate and develop RPD that meet the specific needs of HCP [24].

Our study has a number of limitations. Although it was sufficiently powered to detect pre-defined differences between the control respirators and prototypes, a small sample size in one geographic location limits the generalizability to larger, more diverse populations. Since the participants were aware they were being monitored, their duty performance may have been positively influenced, raising tolerability scores; however, we would expect such an effect to be balanced across randomized arms. [25]; although, the randomized block design should have helped balance this effect across arms. A simulated workplace setting, while faster and less expensive, does not fully reflect the many competing objectives and complexity of a functioning health care system. Similarly, a simulated clinical experience does not precisely reflect the daily occupational responsibilities of HCP who are caring for ill patients. Finally, there are additional forces that may affect adoptability of a new respirator type, such as cost and other market forces, which were not addressed by the outcomes metrics used in this study and should be considered in future studies.

## Conclusion

Compared to the 3M 1870 and 1860, two RPDs commonly used in U.S. health care, tolerability improved for three of four newly developed prototypes in this simulated workplace study. The R-COMFI tool, used in this study to assess tolerability, should be useful for future comparative studies of RPD.

## References

[1] Radonovich L., Cheng J., Shenal B., Hodgson M., & Bender B. (2009). Respirator tolerance in health care workers. JAMA, 301(1), 36–38. 10.1001/jama.2008.894 19126810

[2] Baig A. S., Knapp C., Eagan A. E., & Radonovich L. J. (2010). Health care workers' views about respirator use and features that should be included in the next generation of respirators. American journal of infection control, 38(1), 18–25. 10.1016/j.ajic.2009.09.005 20036443 PMC7132692

[3] Gosch M, Shaffer R, Eagan A, Roberge R, Davey V, Radonovich L. (2013). B95: a new respirator for health care personnel. Am J Infect Control, 41(12), 1224–1230. 10.1016/j.ajic.2013.03.293 23726655 PMC7115300

[4] Institute of Medicine. Preparing for an influenza pandemic: personal protective equipment for health care workers. Washington, DC: The National Academies Press; 2008.

[5] Institute of Medicine. Preparing for an influenza pandemic: personal protective equipment for health care workers update 2010. Washington, DC: The National Academies Press; 2011.

[6] Locatelli S. M., LaVela S. L., & Gosch M. (2014). Health care workers' reported discomfort while wearing filtering face-piece respirators. Workplace health & safety, 62(9), 362–368. 25102476 10.3928/21650799-20140804-03

[7] U.S. Department of Labor, Occupational Safety and Health Administration (OSHA) 29 CFR 1910.134 (1998). Personal protective equipment: user seal check procedures. Available at: https://www.osha.gov/pls/oshaweb/owadisp.show_document?p_table=standards&p_id=9781. Accessed 4/9/2018.

[8] Shenal B. V., Radonovich L.J. Jr., Cheng J., Michael Hodgson M., & Bender B. (2012). Discomfort and exertion associated with prolonged wear of respiratory protection in a health care setting. Journal of Occupational and Environmental Hygiene, 9, pp. 59–64. 10.1080/15459624.2012.635133 22168256 PMC7196691

[9] Bryce E., Forrester L., Scharf S., and Eshghpour M. 2008 What do healthcare workers think? A survey of facial protection equipment user preferences. Journal of Hospital Infection 68(3):241–247. 10.1016/j.jhin.2007.12.007 18295373

[10] Radonovich LJ Jr, Yanke R, Cheng J, Bender B. Diminished speech intelligibility associated with certain types of respirators worn by health care workers. J Occup Environ Hyg. 2010 1;7(1):63–70. 10.1080/15459620903404803 19904661

[11] Kim J., Roberge R., Shaffer R., Zhuang Z., Powell J., Bergman M., & Palmiero A. (2017). Project breathe- Prototype respirator evaluation utilizing newly proposed respirator test criteria. Journal of the International Society for Respiratory Protection, 34, pp. 1–9. PMC10193462 37207040

[12] LaVela S. L., Kostovich C., Locatelli S., Gosch M., Eagan A., & Radonovich L. (2017). Development and initial validation of the Respirator Comfort, Wearing Experience, and Function Instrument [R-COMFI]. Journal of occupational and environmental hygiene, 14(2), 135–147. 10.1080/15459624.2016.1237025 27636378

[13] Cevik MO. (2014) Habituation, sensitization and Pavlovian conditioning. Frontiers in Integrative Neuroscience, 8:13–23 10.3389/fnint.2014.00013 24574983 PMC3920081

[14] Wizner K., Stradtman L., Novak D., and Shaffer R. (2016). Prevalence of respiratory protective devices in U.S. health care facilities. Workplace Health and Safety, 64, pp. 359–369. 10.1177/2165079916657108 27462029 PMC4976391

[15] Radonovich LJ Jr, Bessesen MT, Cummings DA, Eagan A, Gaydos C, Gibert C, Gorse GJ, Nyquist AC, Reich NG, Rodrigues-Barradas M, Savor-Price C, Shaffer RE, Simberkoff MS, Perl TM. The Respiratory Protection Effectiveness Clinical Study (ResPECT): a cluster-randomized comparison of respirator and medical mask effectiveness against respiratory infections in healthcare personnel. BMC Infect Dis. 2016 6 2;16:243 10.1186/s12879-016-1494-2 27255755 PMC4890247

[16] Rebmann T, Carrico R, Wang J. Physiologic and other effects and compliance with long-term respirator use among medical intensive care unit nurses. Am J Infect Control. 2013 12;41(12):1218–23. 10.1016/j.ajic.2013.02.017 23768438 PMC7132714

[17] Carias C, Rainisch G, Shankar M, Adhikari BB, Swerdlow DL, Bower WA, Pillai SK, Meltzer MI, Koonin LM. Potential demand for respirators and surgical masks during a hypothetical influenza pandemic in the United States. Clin Infect Dis.2015 5 1;60 Suppl 1:S42–51. 25878300 10.1093/cid/civ141 PMC7314226

[18] Association of State and Territorial Health Officials Report. Assessment of Respiratory Personal Protective Equipment in U.S. Acute Care Hospitals–2012. November 19, 2014. Available at: http://www.astho.org/Preparedness/Respiratory-PPE-Assessment-Report. Accessed 2/8/2018.

[19] Radonovich LJ, Magalian PD, Hollingsworth MK, Baracco G. Stockpiling supplies for the next influenza pandemic. Emerg Infect Dis. 2009 6;15(6):e1 10.3201/eid1506.081196 21970033 PMC2727308

[20] Baracco G, Eisert S, Eagan A, Radonovich L. Comparative Cost of Stockpiling Various Types of Respiratory Protective Devices to Protect the Health Care Workforce During an Influenza Pandemic. Disaster Med Public Health Prep. 2015 6;9(3):313–8. 10.1017/dmp.2015.12 25874891

[21] Hines S, Mueller N, Oliver M, Gucer P, McDiarmid M. Qualitative Analysis of Origins and Evolution of an Elastomeric Respirator-based Hospital Respiratory Protection Program. Journal of the International Society for Respiratory Protection (2017), 34(2):95–109. 29545673 PMC5849268

[22] Bessesen MT, Adams JC, Radonovich L, Anderson J. Disinfection of reusable elastomeric respirators by health care workers: a feasibility study and development of standard operating procedures. Am J Infect Control. 2015 6;43(6):629–34. 10.1016/j.ajic.2015.02.009 25816692

[23] Bessesen MT, Adams JC, Radonovich L, Anderson J. Disinfection of reusable elastomeric respirators by health care workers: a feasibility study and development of standard operating procedures. Am J Infect Control. 2015 6;43(6):629–34. 10.1016/j.ajic.2015.02.009 25816692

[24] Hines L, Rees E, Pavelchak N. Respiratory protection policies and practices among the health care workforce exposed to influenza in New York State: Evaluating emergency preparedness for the next pandemic. Am J Infect Control, 2014, 42(3): 240–5 10.1016/j.ajic.2013.09.013 24457143 PMC7115259

[25] Chen LF, Vander Weg MW, Hofmann DA, Reisinger HS. The Hawthorne Effect in Infection Prevention and Epidemiology. Infect Control Hosp Epidemiol. 2015 12;36(12):1444–50. 10.1017/ice.2015.216 26383964

